# Pull-Down Into Active Inclusion Bodies and Their Application in the Detection of (Poly)-Phosphates and Metal-Ions

**DOI:** 10.3389/fbioe.2022.833192

**Published:** 2022-03-01

**Authors:** Eva Hrabarova, Martina Belkova, Romana Koszagova, Jozef Nahalka

**Affiliations:** ^1^ Institute of Chemistry, Centre for Glycomics, Slovak Academy of Sciences, Bratislava, Slovakia; ^2^ Institute of Chemistry, Centre of Excellence for White-green Biotechnology, Slovak Academy of Sciences, Nitra, Slovakia

**Keywords:** active inclusion bodies, pyrophosphate, triphosphate, copper, zinc, green fluorescent protein-based biosensors

## Abstract

Inclusion bodies are typically ignored as they are considered unwanted protein waste generated by prokaryotic host cells during recombinant protein production or harmful protein inclusions in human cell biology. However, these protein particles may have applications for *in vivo* immobilization in industrial biocatalysis or as cell-tolerable protein materials for the pharmaceuticals industry and clinical development. Thus, there is a need to *in vivo* “pull-down” (insolubilize) soluble enzymes and proteins into inclusion bodies. Accordingly, in this study, sequences from the short-chain polyphosphatase ygiF were used to design pull-down tags capable of detecting (poly)-phosphates and metal ions. These tags were compared with the entire CHAD domain from *Escherichia coli* ygiF and SACS2 CHAD from *Saccharolobus solfataricus*. The results demonstrated that highly soluble green fluorescent protein variants could be pulled down into the inclusion bodies and could have modified sensitivity to metals and di-/tri-inorganic phosphates.

## Introduction

Inclusion bodies (IBs) are aggregates of partially folded or misfolded proteins. About 15 years ago, despite substantial evidence of their biological functionality (García-Fruitós et al., 2005; Nahálka et al., 2009), IBs were still generally considered inactive/harmful protein wastes produced by mistranslation or other errors in translational and post-translational processes. In prokaryotic host cells, during recombinant protein overexpression, the translational/post-translational apparatus is overloaded and stressed; therefore, IB formation is induced. However, recent reports have shown that enzymes and proteins retain part of their biological activity when aggregated in IBs, and many biotechnological applications of IBs have been proposed, leading to introduction of the term active IBs (aIBs) (Krauss et al., 2017). aIBs have subsequently been shown to have potential applications in the production of recombinant enzymes (Jäger et al., 2020) and have emerged as a solution for *in vivo* enzyme immobilization in industrial biocatalysis (Ölçücü et al., 2021), as demonstrated by pull-down of *Trigonopsis variabilis* d-amino acid oxidase into aIBs (Nahalka and Nidetzky, 2007) and crosslinked inclusion body technology (Nahálka et al., 2008) in industrial biocatalysis. In the pharmaceuticals industry and clinical development, aIBs may have promising applications in the production of antimicrobial peptides (Köszagová and Nahálka, 2020) and nanopills (Villaverde et al., 2015). Indeed, in studies of nanopills, IBs formed using therapeutic proteins may represent a cost-effective protein delivery platform to facilitate the slow release of proteins in mammalian tissues and organs (Villaverde et al., 2015). Despite the serious limitations of this approach, including the bacterial origin of IBs and undefined fraction of bacterial host cell proteins in IBs, many researchers have started to focus on the potential medical applications of IBs. For example, aIBs of *Pseudomonas aeruginosa* exotoxin show potential for selective destruction of primary tumor tissues in mouse models of human colorectal cancer (Céspedes et al., 2020). In addition to applications in industrial biocatalysis and pharmaceutical development, IBs could also be used as biosensors. Indeed, IBs could be used for direct biosensing of various compounds in mammalian tissues and organs. In addition, the first experiments purposely exploring functionality of IBs were successfully done on fluorescence emission of aIBs, on the fluorescent proteins fused to different aggregating polypeptides (García-Fruitós et al., 2005). Notably, suspensions of purified green fluorescent protein (GFP)/blue fluorescent protein IBs showed 20%/30% fluorescence activity relative to that of soluble protein (García-Fruitós et al., 2005). On the other hand, there is a broad scope of the potential application of fluorescent aIBs; FRET-biosensors based on a fluorophore protein pair are very well established in the clinical, pharmaceutical, toxicological, or agri-food analysis (Zhang et al., 2019).

**FIGURE 1:**
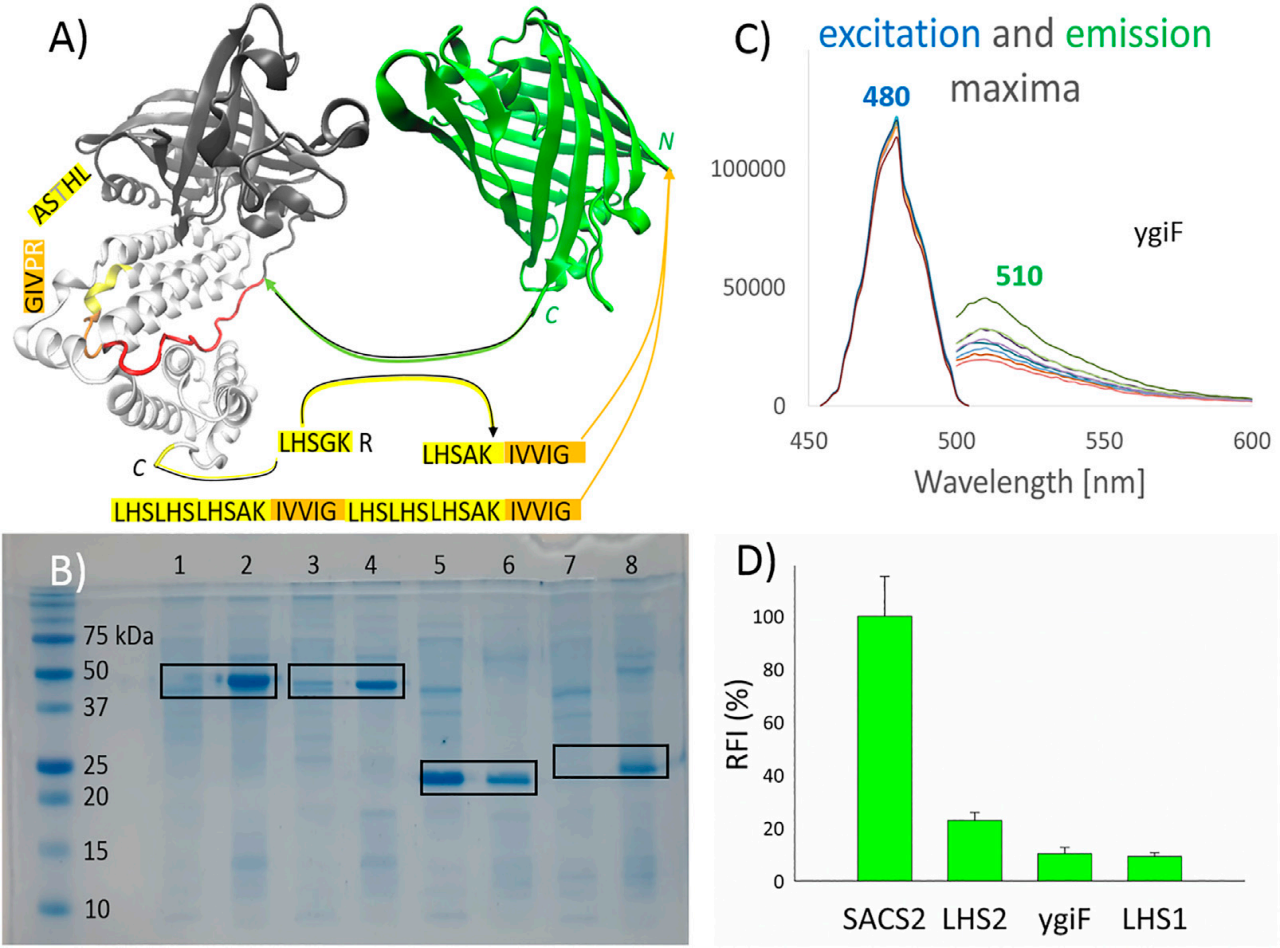
Pull-down into active inclusion bodies. **(A)** ygiF inorganic (poly)-phosphatase from *Escherichia coli*. Tunnel metalloenzyme catalytic domain (TTM, black); polyP binding CHAD domain (white); and the used linker (red) are shown. TurboGFP (green), with its C*-*terminus connected to the linker. PolyP recognition sequences (yellow) were fused with a minimum hydrophobic sequence (orange) and attached to the N*-*terminus of turboGFP. The graphical illustrations and the sequences of the constructs are shown in the supplementary information (Supplementary Material). **(B)** Sodium dodecyl sulfate polyacrylamide gel electrophoresis. Lanes 1/2, SACS2 construct supernatant/sediment; lanes 3 and 4, ygiF construct supernatant/sediment; lanes 5 and 6, LHS1 construct supernatant/sediment; lanes 7 and 8, LHS2 construct supernatant/sediment. The supernatant of the cell lysate and the washed sediment of the cell lysate (aIBs) were diluted equally. **(C)** The excitation and emission maxima of ygiF aIBs (PPi gradient, no metal presence). **(D)** The relative fluorescence activity (RFI) of aIBs.

Polyphosphate (polyP) is an energy source and active metabolic regulator (Achbergerová and Nahálka, 2011). Hydrolysis of polyp leads to the generation of nucleotide triphosphate, triphosphate, pyrophosphate, phosphate, and the other phosphorylation products. Histidine α-helical domains were recently identified as specific polyP-binding modules; for example, a small helical domain at the C-terminus of the bacterial short-chain polyphosphatase ygiF, previously annotated as conserved histidine α-helical domain (CHAD), has been confirmed to be a polyP-binding module (Lorenzo-Orts et al., 2019). The catalytic domain of *Escherichia coli* ygiF polyphosphatase has been evaluated as an example of a triphosphate tunnel metalloenzyme (TTMs), which are present in all kingdoms of life and catalyze diverse enzymatic reactions, such as mRNA capping, the cyclization of adenosine triphosphate, the hydrolysis of thiamine triphosphate, and the synthesis and breakdown of inorganic polyphosphates (Martinez et al., 2015). ygiF polyphosphatase has been crystalized, and the full-length structure has been reported (Figure 1A) (Martinez et al., 2015).

Several aggregation-prone or “pull-down” tags have been established to produce active recombinant enzymes in the form of aIBs; in our laboratory, a 20-kDa cellulose-binding domain from *Clostridium cellulovorans* (CBDclos) is used for this purpose. Moreover, many aggregation-prone small proteins exist that effectively sequester highly soluble recombinant polypeptides into aIBs (de Groot et al., 2009; Gil-Garcia and Ventura, 2021); however, the most attractive approach is to use small aggregating peptides (Wu et al., 2011; Zhou et al., 2012; Wang et al., 2015; Carratalá et al., 2021). The surfactant-like peptides L6K2 (Carratalá et al., 2021), ELK16 (Wu et al., 2011), L6KD (Zhou et al., 2012), and GFIL8 hydrophobic tag (Wang et al., 2015) have been shown to yield the best “pull-down” results. However, ideal pull-down tags with both surfactant-like properties and a hydrophobic tag have not been reported. On the other hand, insolubilizing nona-peptide (GILQINSRW) was applied as “pull-down” tag for insoluble expression of highly soluble halophilic metal binding protein for metal ion biosorption (Tokunaga et al., 2019) and various polar metal binding peptides were engineered in *Escherichia coli* cells to recovery metals from polluted wastewater (Maruthamuthu et al., 2015a; Maruthamuthu et al., 2015b; Maruthamuthu et al., 2018), so in the close future, aIBs based on aggregation-prone and metal binding/sensing peptides will be probably designed. The aIBs can be also produced by small coiled-coil domains, a natural coiled-coil proteins promote nonamyloid supramolecular interactions, so these non-toxic IBs may better preserve the activity of the attached enzymes or the other polypeptides (Diener et al., 2016; Gil-Garcia et al., 2020).

Several strategies have been used for the design of GFP-based sensors. For example, in FRET analysis, the ligand binding domain is often inserted between a cyan fluorescent protein (CFP) and a yellow fluorescent protein (YFP). Alternatively, a single fluorescent protein is split into two parts, which are then individually attached to the ends of a ligand binding domain. For example, various peptide sequences inserted between CFP and YFP were tested for glucose binding/sensing (Nahalka and Hrabarova, 2021), and citrine (YFP) has been split into two parts, which were then attached to the ends of the glucose binding domain and effectively used for sensing of glucose (Mita et al., 2019). The basic advantage of single fluorescent protein-type indicators is that they only require acquisition of one emission signal, whereas FRET protein-type indicators require acquisition of two emission signals (Deng et al., 2020).

In this study, we aimed to design peptides for “pull-down” into aIBs and their direct application in the detection of (poly)-phosphates and metal ions using a single fluorescent protein-type approach and a nonaggregating and extremely rapidly maturating GFP variant (turboGFP) (Evdokimov et al., 2006).

## Materials and Methods

### Cloning and Expression

The amino acid sequences encoded by the genes used in this study and the amino acid sequences of the fusion GFP constructs are shown in the supplementary information (Supplementary Material). Codon optimization for *E. coli*, gene synthesis, and cloning (*Nde*I/*Bam*HI) with the pET-30b (+) plasmid were performed using GenScript. A terminating stop codon was added to the insert; therefore, no His-tag was included. Chemically competent *E. coli* BL21 (DE3)T1R cells were transformed with the plasmids and cultivated on kanamycin/LB/agar plates. The colonies were regrown in 30 ml LB medium supplemented with kanamycin (30 μg/ml) for approximately 20 h at 32°C; then, 15 ml of the solution was transferred to a 300-ml flask with 100 ml LB-media and incubated for 4 h at 37°C with shaking at 150 rpm. Recombinant expression was accomplished by adding an inductor (isopropyl β-d-1-thiogalactopyranoside; 400 μM) for 20 h at 15–20°C with shaking at 150 rpm. After centrifugation (45 min, 9700 × *g*, 4°C), the biomass was suspended in a minimum volume of water, frozen, and immediately lyophilized. The lyophilized cells were kept at −30°C until further use.

### Isolation of IBs

Ten milligrams of lyophilized cells was lysed twice using 500 µL BCL (CelLytic B Cell Lysis Reagent; Sigma-Aldrich, MO, United States). After centrifugation of the lysate (5 min, 21,000 × *g*, 4°C), cell debris was washed three to five times using 500 µL Tris-HCl buffer (50 mM, pH 7.5). After the final centrifugation step, IBs were resuspended in 500 µL Tris buffer and stored at −30°C. The IBs were then used at a 100-fold dilution.

### Sodium Dodecyl Sulfate Polyacrylamide Gel Electrophoresis

We prepared 12% separation gels and 4% stacking gels in our laboratory and allowed the gels to stand for approximately 3 h to achieve optimal polymerization. Before loading on the gel, samples were mixed with Laemmli buffer containing β-mercaptoethanol and heated at 75°C for 15 min. The prepared gels were washed, stained with Colloidal Coomassie Stain overnight, and thoroughly washed again. Band intensity was evaluated using UVP Doc-It LS Analysis Software (ThermoFisher Scientific, MA, United States) to determine the percentage of the recombinant protein in the cleared cell lysate and in the suspension of IBs.

### Fluorescence Measurement

Measurement of fluorescence was performed using a Mithras^2^ LB 943 Multiplate reader (Berthold Technologies GmbH & Co. KG, Bad Wildbad, Germany). Samples in Tris-HCl buffer (50 mM, pH 7.5) were loaded into black 96-well plates with transparent well bottom (cat. no. 781671; BRAND plates, Wertheim, Germany). The samples were mixed by pipetting, and fluorescence was measured 30 min later in duplicate using an excitation filter at 485 nm and an emission filter at 535 nm. Measured values were evaluated using the MAVE (average of multiple measurement for single well) function. In Figure 1D, the RFI mean values and standard errors are calculated from eight replicates; in the other figures, the FI values and standard errors are taken from the duplicate samples and “zero points” (no metals or phosphates) are calculated from triplicates at least. The excitation and emission maxima were measured on a Tecan Infinite 200 Microplate Reader.

## Results and Discussion

### Pull-Down Into aIBs

Enzymes and other proteins can be effectively immobilized *in vivo* by the fusion of an aggregation-prone module/tag to a target recombinant protein. In our laboratory, we used 20-kDa CBDclos, which was originally proposed as a cellulose affinity tag, and was *N*-terminally inserted in front of the cloning site of the commercial Novagen vector pET-34b (+). However, we found that the globular cellulose binding domain acted like a strong aggregation-prone module/tag. Therefore, this domain was linked to the target recombinant protein using pET-34b (+) DDDK-enterokinase-SPG linker, and the 95–100% volumetric activity of the fused enzyme was effectively pulled down into aIBs (Nahalka and Nidetzky, 2007; Nahálka et al., 2008). When we expressed His/Strep-tagged soluble forms of the same enzymes, despite the higher specific activities (U/g of protein) of the purified soluble proteins, aIBs were produced in the cells until the volumetric activity (U/L of fermentation broth) was the same as that of the soluble proteins, highlighting the attractiveness of aIBs as immobilized enzymes. In addition, the use of aIBs technologically simplifies the isolation protocols for recombinant enzymes comparing with affinity-based isolation procedures commonly used for soluble enzymes. In summary, targeted protein expression to aIBs means “pull-down”. CBDclos offers satisfactory pull-down results into aIBs and normally shows little interference with the folding and function of its fusion partners (Nahalka and Nidetzky, 2007; Nahálka et al., 2008). However, aggregation-prone short peptides have generally been selected for pull-down of recombinant enzymes/proteins into aIBs (Wu et al., 2011; Zhou et al., 2012; Wang et al., 2015; Carratalá et al., 2021).

Accordingly, in this study, we attempted to design small aggregation-prone peptides with selectivity for (poly)-phosphates. YgiF inorganic (poly)-phosphatase from *E. coli*, which exhibits a conserved mechanism for triphosphate and metal cofactor binding (Martinez et al., 2015), was selected for this study. The chemistry of polyP involves appropriate coordination of polyP^n−^ and Me^2+^ ions, and the catalytic TTM of ygiF polyphosphatase (Figure 1A, black) as well as CHAD (Figure 1A, white), which is attached through a linker (Figure 1A, red), act together to fulfill this function. In ygiF polyphosphatase, the CHAD enhances enzymatic activity and may be used to engineer polyP-metabolizing enzymes and specifically localize polyP stores in eukaryotic cells and tissues (Lorenzo-Orts et al., 2019). In our study, we cleaved off the TTM (Figure 1A, black) and attached the C-terminus of turboGFP to the linker (Figure 1A, red), which was ligated to the CHAD (Figure 1A, white). No affinity purification tag was used; instead, a small 10-amino acid peptide (LHSAKIVVIG) was applied, and the “pull-down” tag was fused to the N-terminus of turboGFP. The LHSGKR C-terminal short sequence of ygiF polyphosphatase was not highly conserved (not shown data); however, we hypothesized that this sequence was a short or minimal sequence required for recognition of polyP^n−^ and Me^2+^ ions. A similar sequence, C-LHTSAR, was found in the N-terminus of the CHAD. Using a combination of these sequences, we designed the LHSAK minimal sequence (Figure 1A, yellow). A minimum hydrophobic GFIL peptide, which could form gel-phase materials *via* self-assembly, was previously doubled to a GFILGFIL octapeptide and successfully used for pull-down into aIBs (Wang et al., 2015). In ygiF polyphosphatase, C-LHTSAR continued into a VIG hydrophobic triplet, and we doubled the hydrophobic pair to the IVVIG sequence and fused this sequence with the proposed LHSAK minimal sequence. The final 10-amino acid LHSAKIVVIG peptide (LHS1) was used as the minimum pull-down tag sequence that could recognize polyP^n−^ and Me^2+^ ions. To ensure that the tag would recognize the ions and pull down the construct effectively, we triplicated the LHS sequence and doubled the hydrophobic core, yielding a second 32-amino acid tag ((LHS)_3_AKIVVIG)_2_ (LHS2). Q97YW1_SACS2, an uncharacterized protein from *Saccharolobus solfataricus*, was recently described as a small CHAD that bound polyP more tightly than ygiF/CHAD (Lorenzo-Orts et al., 2019). In the SACS2 construct, the 10-amino acid LHSAKIVVIG peptide was fused to the N-terminus of the SACS2/CHAD, and the construct was attached to the N-terminus of turboGFP. The same linker as that used for ygiF construct (IKPTTILHVAAKAD) was applied; however, the SACS2/CHAD domain became N-terminally fused. Overall, we obtained four turboGFP fusion constructs for subsequent testing.

Figure 1B shows the pull-down results. Sodium dodecyl sulfate-poly acrylamide gel electrophoresis was used to compare soluble and insoluble fractions of cell lysates, with equal dilution of supernatants and sediments. As demonstrated in the figure, LHS1-GFP-ygiF/CHAD, LHS1-SACS2/CHAD-GFP, and LHS2-GFP were effectively “pulled-down” into aIBs (90–100%), whereas LHS1-GFP remained almost 60% soluble, as determined using band intensity analysis software. TurboGFP exhibits extremely fast protein-folding kinetics and chromophore maturation, with high protein solubility (Evdokimov et al., 2006). Thus, it was unsurprising that the small hydrophobic core in the 10-amino acid LHS1 was able to be pulled-down into IBs at a range of only approximately 40% of total LHS1-GFP. By contrast, the doubled hydrophobic core inside the ((LHS)_3_AKIVVIG)_2_ 32-amino acid tag LHS2 was sufficient for “pull-down” purposes. Nevertheless, LHS1 tag worked effectively when the solubility of the turboGFP was lowered by the N-terminal fusion of SACS2/CHAD or by the C-terminal fusion of ygiF/CHAD (Figure 1B).

Figure 1D compares the fluorescent activities of the designed constructs. LHS1-SACS2/CHAD-GFP fusion protein showed almost one magnitude higher fluorescence than LHS1-GFP-ygiF/CHAD. These results were logical because in the LHS1-SACS2/CHAD-GFP fusion protein, turboGFP was separated from the LHS1 tag and SACS2/CHAD by a natively designed linker from ygiF polyphosphatase (IKPTTILHVAAKAD), and the direct N-terminal fusion with LHS2 and LHS1 tags lowered the florescence by 5 and 10 times, respectively. C-terminal fusion of CHAD through the linker had no increasing negative effect. Thus, separation of the aggregation-prone tags by a disordered “noninteractive” linker could be used for the construction of enzymatically/physiologically active IBs. However, incomplete protein folding and direct fusion without a linker could be advantages when extreme sensitivity is required, e.g., in the case of “turn-off” biosensors. In the fused GFP, the excitation and emission maxima could be shifted; however, in this study, all tested fusion proteins had the same excitation maximum (480 nm) and the same emission maximum (510 nm; Figure 1C).

In summary, when attempting to pull down highly soluble proteins using short LHS1/LHS2 tags, it may be preferable to use the LHS2 tag or fuse the construct with another protein domain. If the recombinant protein can partially enter IBs, then LHS1 tag may be sufficient for 90–100% pull down into IBs.

### Detection of Metal Ions

**FIGURE 2:**
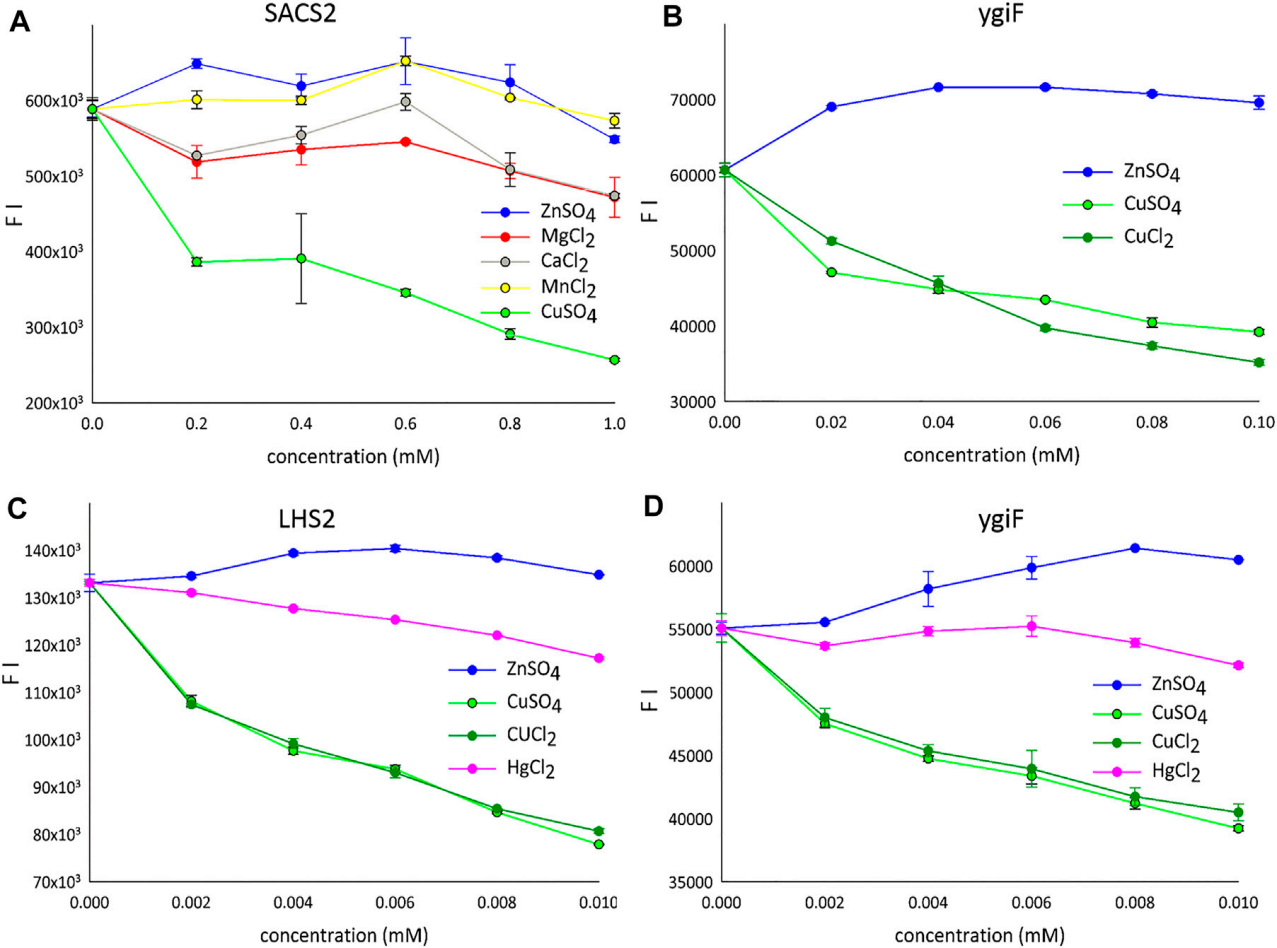
The detection of metal ions. **(A)** LHS1-SACS2/CHAD-turboGFP fusion construct (range: 0.2–1 mM). **(B)** LHS1-turboGFP-ygiF/CHAD fusion construct (range: 0.02–0.1 mM). **(C)** LHS2-turboGFP fusion construct (range: 0.002–0.01 mM). **(D)** LHS1-turboGFP-ygiF/CHAD fusion construct (range: 0.002–0.01 mM). FI means the fluorescence intensity and only one protein is shown for the each concentration gradient.

Complex analytical instruments, such as atomic absorption or inductively coupled plasma mass spectrometers, are required for sensitive detection of transition metals in environmental and biological samples. Electrochemical methods require simpler instruments but suffer from a lower detection sensitivity (Bansod et al., 2017). Therefore, protein-, RNA-, and DNA- based sensors have been introduced for various metal ions. In particular, metal ion-quenchable fluorescent proteins that are selectively modulated by various Me^2+^ ions have been developed as biosensors. For example, a histidine-modified GFP was engineered to sense 10 nM Cu^2+^ ions (Bálint et al., 2013), and selective protein unfolding was applied to differentiate metal ions and produce metal-specific fluorescence intensity (FI) signals (Sorenson and Schaeffer, 2020). A metal-binding biotin protein ligase fused to GFP, when coupled with differential scanning fluorimetry, was shown to yield distinct protein unfolding signatures with Zn^2+^ and Cu^2+^ ions in aqueous solutions (Sorenson and Schaeffer, 2020). Interestingly, we observed similar results; incompletely folded fusion variants of turboGFP in the form of aIBs showed a tendency for increased FI for Zn^2+^ ions and markedly decreased FI for Cu^2+^ ions under optimal conditions (pH 7.5, 20°C, Figure 2). Figure 2A shows fluorescence quenching by various Me^2+^ ions tested using the SACS2 fusion construct (SACS2). When the concentration was 1 mM lower, only Cu^2+^ ions quenched the fluorescence by more than 50%. The other tested metals showed weak quenching of fluorescence at approximately 1 mM; however, when lower concentrations were used, weak increases in fluorescence were observed (0.2 mM Zn^2+^, 0.6 mM Mn^2+^), and calcium and magnesium showed local maxima at 0.6 mM after a gradual initial decrease in fluorescence. In summary, only Cu^2+^ ions are well discriminated from the other metals. Presented on the ygiF construct (ygiF), Figure 2B shows the fluorescence “window” between Zn^2+^ and Cu^2+^ ions and compares the influence CuSO_4_ and CuCl_2_ compounds. There were minor differences between the presence of SO_4_^2-^ and Cl^−^ ions, although no differences were observed in the range of 0.002–0.01 mM (Figures 2C,D). It means that FI depends only on metal cations (Cu^2+^) and is not influenced by the anions of the salts (Cl^−^/SO_4_^2−^).

Mercury (Hg) is a global pollutant and poisonous metal, adversely affecting human health and the surrounding environment. Hg toxicity depends on the chemical form; Hg° is considered nontoxic, whereas Hg^2+^ is the most toxic form. Therefore, many GFP-dependent Hg^2+^ biosensors have been developed to date (Kumari et al., 2020). A chemosensor designed and synthesized based on the GFP-chromophore showed a visible Zn^2+^-specific fluorescence turn-on reaction and Hg^2+^/Cu^2+^-specific fluorescence turn-off reaction (Shi et al., 2012). Accordingly, we next investigated the influence of HgCl_2_ on the fluorescence of the designed constructs. However, Hg^2+^ ions quenched the fluorescence very weakly compared with Cu^2+^ ions (Figures 2C,D).

**FIGURE 3:**
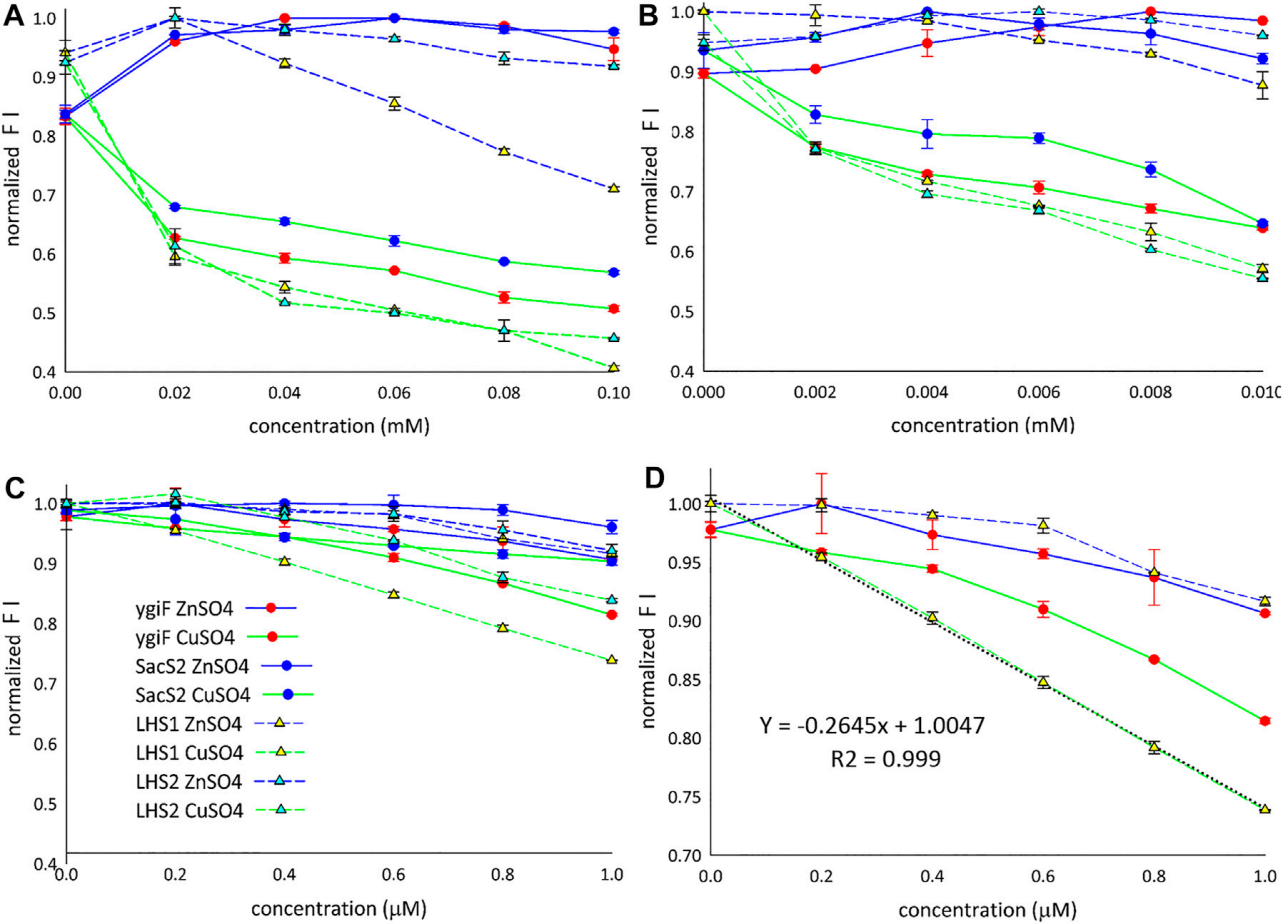
Detection of Cu^2+^ and Zn^2+^ ions. **(A)** Range: 0.02–0.1 mM. **(B)** Range: 0.002–0.01 mM. **(C)** Range: 0.2–1 µM. **(D)** LHS1 construt linear correlation (range: 0–1 µM). FI means the fluorescence intensity.

Figure 3 shows the fluorescence “window” between Zn^2+^ and Cu^2+^ ions for all four designed constructs. The detection limit for Cu^2+^ ions was found to be approximately 1 µM (Figure 3C), and only LHS1 construct adequately distinguished between the compared metal ions at concentrations lower than 1 µM (Figure 3D). We also observed an exceptional linear correlation between LHS1 construct and Cu^2+^ ions within this concentration range (Figure 3D). The LHS1-quenching ability of turboGFP was also different in the range of 0.02–0.1 mM (Figure 3A). Therefore, these findings suggest that the sensitivity of LHS1 construct could be an effect of the direct fusion of the LHS1 tag to the N-terminus of turboGFP. However ygiF construct, which is an LHS1 construct plus a C-terminal CHAD, did not show this sensitivity (Figures 3A,D). It is possible that the CHAD buffered the Cu^2+^ ions in this case.

Overall, these results showed that Zn^2+^ ions slightly increased the fluorescence of the designed constructs and that Cu^2+^ ions markedly decreased the fluorescence of the designed constructs. LHS1 construct was somehow an exception; turn-off of the fluorescence response was sensitive and linear (Figures 3A,D). Despite the general trend that Zn^2+^ turned on and Cu^2+^ turned off specific fluorescence responses, all four constructs showed slightly different curves, indicating that the fusion tags/domains differentially influenced GFP fluorescence. *E. coli* ygiF and *S. solfataricus* SACS2 CHADs contain conserved histidine α-helical domains, and the polyhistidine LHS2 tag could be included in one group, although LHS1 construct was unique. Thus, incomplete folding of IBs and direct N-terminal fusion of the LHS1 tag may enhance the quenching sensitivity of turboGFP. By contrast, *E. coli* ygiF, *S. solfataricus* SACS2 CHADs, and the “polyhistidine” LHS2 tag may interact with Cu^2+^ ions and guard the chromophore, consistent with the observation that quenching was not observed at concentrations less than 1 µM.

### Detection of (Poly)-Phosphates

Inorganic polyphosphates are primitive in their origin and act as a reservoir for inorganic phosphate and energy (macroergic bonds). These molecules also directly regulate various vital processes in the cell and are have roles in gene regulation. Energy metabolism and regulation are orchestrated by polyphosphate kinases (PPKs) (Achbergerová and Nahálka, 2011). In our laboratory, we have focused on multi-enzymatic reactions powered by polyP and PPKs, leading to the synthesis of poly/oligosaccharides (Nahálka and Pätoprstý, 2009). In poly/oligosaccharide enzymatic chemistry, Mg^2+^ (at concentrations above 20 mM) or Mn^2+^ (at concentrations above 5 mM) must be present in the reaction mixture as cofactors for glycosyltransferases, for the recognition of monosaccharide substrates marked by a nucleoside diphosphate. Nucleoside diphosphate sugars are synthetized from the nucleoside triphosphates and monosaccharide 1-phosphates by pyrophosphorylases, which also use these metal cofactors for recognition of nucleoside di-/tri-phosphates (Nahalka et al., 2003). In the reaction, pH (7.5–8.0) and the concentrations of nucleoside di-/tri-phosphates and inorganic Pi, PPi, and PPPi are tightly controlled to avoid precipitation of magnesium and manganese phosphate salts.

**FIGURE 4:**
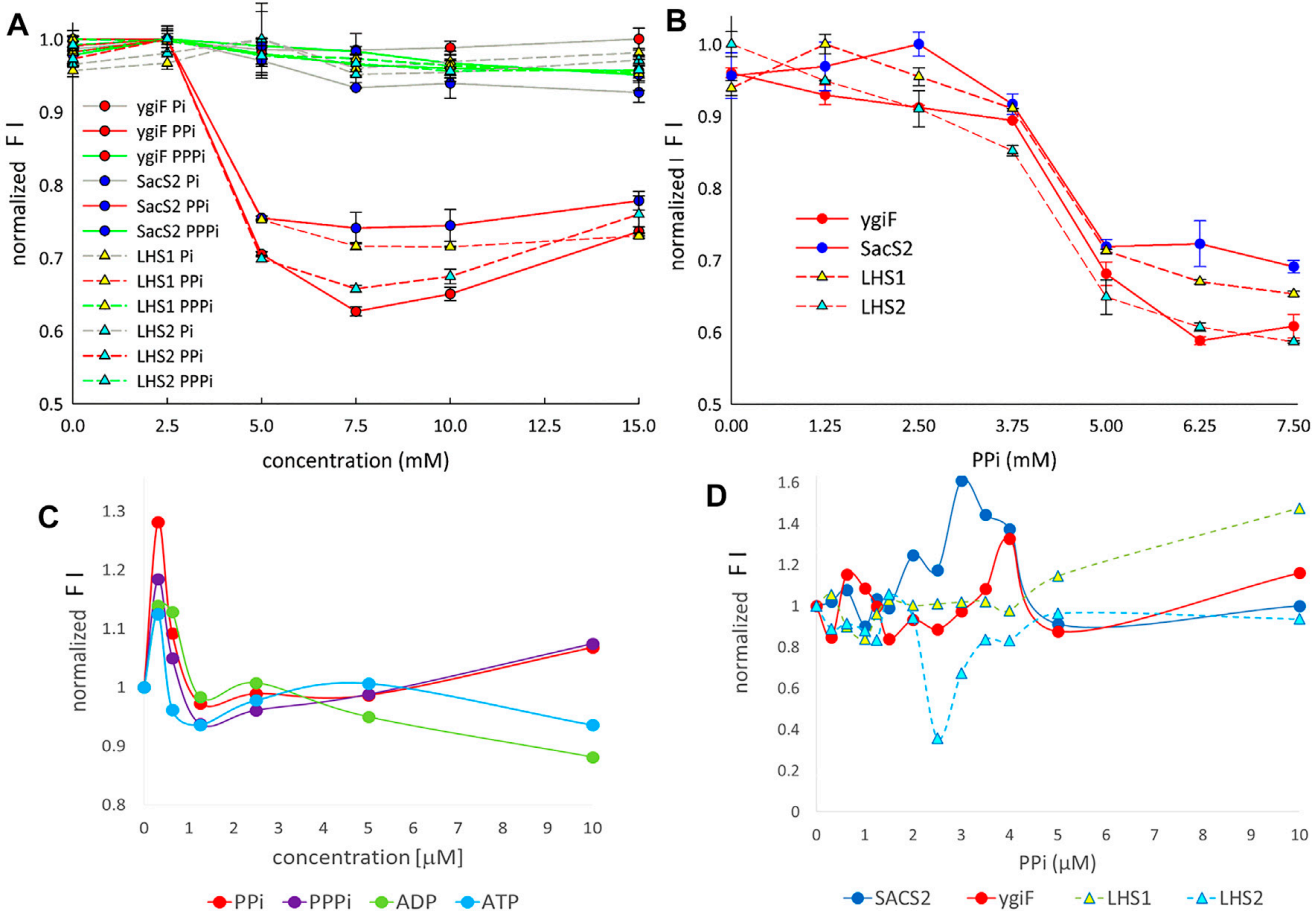
The detection of (poly)-phosphates. **(A)** The influence of Pi/PPi/PPPi on the relative fluorescence intensity in the range from 0 to 15 mM and in the presence of 20 mM Mg^2+^ (measured at 30 min after mixing phosphates with magnesium and consequent mixing with GFP-IBs). **(B)** Calibration in the presence of 20 mM Mg^2+^, which mimicked PPi (7.5 mM) hydrolysis to 2Pi (15 mM). **(C)** Influence of PPi/PPPi and ADP/ATP on the SACS2 FI in the range from 0 to 10 µM and in the presence of 1.5 mM Pi but without Me^2+^ ion. **(D)** Influence of PPi on the FI in the range from 0 to 10 µM and in the presence of 1.0 mM Mg^2+^, 1.4 mM Ca^2+^, and 1.5 mM Pi. For c and d, the results were obtained from the exact emission maxima (506–512 nm) from spectral scans. FI means the fluorescence intensity.

Therefore, in this study, we next attempted to obtain a IB-biosensor that could monitor (poly)-phosphates in the reaction mixture. Unfortunately, we did not find reproducible conditions for the on-line monitoring of PPi and nucleoside triphosphates by turboGFP aIBs in the reaction mixtures. However, the sampling and discontinuous measurement of the fluorescence intensity (FI) under precise pH and timing conditions yielded reproducible results. As shown in Figure 4A, Pi and PPPi did not influence FI in the range from 0 to 15 mM in the presence of 20 mM Mg^2+^. By contrast, PPi quenched the FI by approximately 30% in the range from 5 to 15 mM under the same conditions. Figure 4B shows the mimicking of PPi (7.5 mM) hydrolysis by a phosphatase; zero PPi indicates the presence of 15 mM Pi or 5 mM PPi indicates the presence of 5 mM Pi. The results in Figure 4B clearly corresponded with those in Figure 4A, suggesting that Pi did not influence the FI and that the curve may be used as a calibration curve for PPi hydrolysis. For example, the LHS2 fusion protein showed a linear decrease in FI from 0 to 3.75 mM. The IBs could be easily produced and isolated, and aIBs based on turboGFP could have potential applications in inexpensive, rapid detection of PPi in various samples. Sodium PPi (E450) and PPPi (E451) are used widely as relatively safe food additives (sequestrants and water retention agents) and as additives in commercial detergents (water softeners). By contrast, PPi has pivotal roles in many physiological reactions, is a known biomarker for various diseases, including cancer and infectious diseases, and is an essential target in diverse fields, including ecological research (Anbu et al., 2021). Therefore, many Zn^2+^- and Cu^2+^-based fluorescent sensors have been developed for the detection of PPi (Anbu et al., 2021).

Based on these sensors, we next explored whether our constructs could be used to sense PPi in plasma. Interestingly, the fusion proteins were sensitive to micromolar concentrations of (poly)-phosphates; for example, SACS2 construct turned-on the fluorescence response at concentrations under 1 µM and was able to distinguish between PPi/PPPi and ADP/ATP at a concentration of 10 µM (Figure 4C). The concentration of human plasma PPi in normal individuals is 2.63 ± 0.47 μM (O'Neill et al., 2010). ATP, ADP, and AMP have been detected in conventional clinical samples at concentrations of at least 1 µM (Harkness et al., 1984). However, when ATP is not released from the blood during plasma preparation, the concentration is much lower (Gorman et al., 2007). Importantly, the measurements were not performed directly in the plasma, but instead in 50 mM Tris buffer containing 1.5 mM Pi but lacking Me^2+^. The reference interval for Pi in plasma is 0.8–1.5 mM (Bazydlo et al., 2014). Na^+^, Ca^2+^, and Mg^2+^ are the main positive plasma electrolytes, with reference intervals of 0.7–1.1 mM for magnesium and 1.1–1.4 mM for free, physiologically active calcium (Bazydlo et al., 2014). When 50 mM Tris buffer containing 1.5 mM Pi was supplemented with 1.0 mM Mg^2+^ and 1.4 mM Ca^2+^, the main plasma metals chelated by PPi, the fluorescence turn-on peak was shifted to 3 μM in the case of SACS2 construct (Figure 4D). In the case of ygiF construct, the FI maximum was 4 μM. Interestingly, the LHS2 fusion protein exhibited a reverse peak, with a minimum at 2.5 μM. The LHS1 construct showed a plateau in the range from 1.5 to 4 μM, suggesting that fusion with the small 10-amino acid LHS1 aggregating peptide was less sensitive to PPi. Although this seemed logical, measurements at 5 and 10 μM indicated that the construct was still sensitive to PPi and that the peak could shift to this range (Figure 4D).

In summary, our preliminary experiments suggested that the constructs may have potential applications in the detection of PPi in the plasma. Further studies are needed to assess real plasma samples.

## Conclusion

IBs are aggregates of partially folded or misfolded proteins. Initially, IBs were considered inappropriate for spectral measurement because IB suspensions sediment over time and the dispersion of light is influenced by nano-sized particles, such as IBs. However, IBs are relatively inexpensive biological materials, and incomplete protein folding can be an advantage in the development of sensitive biosensors, particularly when in quenching mode. GFP-based biosensors are commonly used in clinical, pharmaceutical, toxicological, and agri-food analyses, and future studies of the applications of IBs in these fields would be interesting.

Bacterial, archaeal, and eukaryotic CHADs have been identified only recently as specific polyP-binding modules. Accordingly, CHADs have been proposed to modulate the properties of polyP-metabolizing enzymes and specifically localize polyP stores in eukaryotic cells and tissues (Lorenzo-Orts et al., 2019). *E. coli* ygiF polyphosphatase is an example of a triphosphate tunnel metalloenzyme that uses a CHAD to enhance its activity. The CHAD of ygiF contains a C-terminal LHSGKR sequence and a similar sequence on its N-terminus (C-LHTSAR); our findings suggested that these sequences may be the minimum sequence required for recognition of polyP. Modification to LHSAK and fusion with the minimal aggregation-prone sequence IVVIG enabled us to design a minimal aggregation tag that recognizes (poly)-phosphates and metal ions. Using this LHS1 tag in the N-terminus of the fusion constructs may effectively “pull-down” the activity into aIBs for proteins that are not highly soluble (SACS2 and ygiF fusions). Although the pull-down effect is markedly decreased for highly soluble proteins (turboGFP), the double IVVIG core in the LHS2 tag completely shifted highly soluble turboGFP into aIBs (Figure 1B). In light of fluorescect or enzymatic activity, it is important to use a spacer/linker between the N-terminal LHS1/2 tag and enzyme because the SACS2 construct, which contained a natively designed IKPTTILHVAAKAD linker, showed 10-fold higher fluorescence than LHS1 and ygiF fusions. However, for quenching-mode GFP-biosensors, fusion without a linker may be advantageous because the LHS1 construct is highly sensitive to Cu^2+^ quenching. More importantly, fusion proteins may be sensitive to micromolar concentrations of (poly)-phosphates.

In the implications of the findings, aIBs can be considered as the naturally occurring nanoparticles in most cell types that could be used as the regular GFP-based biosensor applicable in clinical, pharmaceutical, toxicological, and agri-food analyses.

## Data Availability

The original contributions presented in the study are included in the article/Supplementary Material, further inquiries can be directed to the corresponding author.

## References

[B1] Achbergerová L. Nahálka J. (2011). Polyphosphate - an Ancient Energy Source and Active Metabolic Regulator. Microb. Cel Fact. 10, 63. 10.1186/1475-2859-10-63 PMC3163519 21816086

[B2] Anbu S. Paul A. Stasiuk G. J. Pombeiro A. J. L. (2021). Recent Developments in Molecular Sensor Designs for Inorganic Pyrophosphate Detection and Biological Imaging. Coord. Chem. Rev. 431, 213744. 10.1016/j.ccr.2020.213744

[B3] Bálint E.-É. Petres J. Szabó M. Orbán C.-K. Szilágyi L. Ábrahám B. (2013). Fluorescence of a Histidine-Modified Enhanced green Fluorescent Protein (EGFP) Effectively Quenched by Copper(II) Ions. J. Fluoresc. 23, 273–281. 10.1007/s10895-012-1145-y 23129167

[B4] Bansod B. Kumar T. Thakur R. Rana S. Singh I. (2017). A Review on Various Electrochemical Techniques for Heavy Metal Ions Detection with Different Sensing Platforms. Biosens. Bioelectron. 94, 443–455. 10.1016/j.bios.2017.03.031 28340464

[B5] Bazydlo L. A. L. Needham M. Harris N. S. (2014). Calcium, Magnesium, and Phosphate. Lab. Med. 45, e44–e50. 10.1309/LMGLMZ8CIYMFNOGX

[B6] Carratalá J. V. Cisneros A. Hellman E. Villaverde A. Ferrer-Miralles N. (2021). Title: Insoluble Proteins Catch Heterologous Soluble Proteins into Inclusion Bodies by Intermolecular Interaction of Aggregating Peptides. Microb. Cel Fact. 20, 30. 10.1186/s12934-021-01524-3 PMC7852131 33531005

[B7] Céspedes M. V. Cano‐Garrido O. Álamo P. Sala R. Gallardo A. Serna N. (2020). Engineering Secretory Amyloids for Remote and Highly Selective Destruction of Metastatic Foci. Adv. Mater. 32, 1907348. 10.1002/adma.201907348 31879981

[B8] de Groot N. S. Sabate R. Ventura S. (2009). Amyloids in Bacterial Inclusion Bodies. Trends Biochem. Sci. 34, 408–416. 10.1016/j.tibs.2009.03.009 19647433

[B9] Deng H. Yan S. Huang Y. Lei C. Nie Z. (2020). Design Strategies for Fluorescent Proteins/mimics and Their Applications in Biosensing and Bioimaging. Trac Trends Anal. Chem. 122, 115757. 10.1016/j.trac.2019.115757

[B10] Diener M. Kopka B. Pohl M. Jaeger K.-E. Krauss U. (2016). Fusion of a Coiled-Coil Domain Facilitates the High-Level Production of Catalytically Active Enzyme Inclusion Bodies. ChemCatChem 8, 142–152. 10.1002/cctc.201501001

[B11] Evdokimov A. G. Pokross M. E. Egorov N. S. Zaraisky A. G. Yampolsky I. V. Merzlyak E. M. (2006). Structural Basis for the Fast Maturation of Arthropoda green Fluorescent Protein. EMBO Rep. 7, 1006–1012. 10.1038/sj.embor.7400787 16936637 PMC1618374

[B12] García-Fruitós E. González-Montalbán N. Morell M. Vera A. Ferraz R. M. Arís A. (2005). Aggregation as Bacterial Inclusion Bodies Does Not Imply Inactivation of Enzymes and Fluorescent Proteins. Microb. Cel Fact. 4, 27. 10.1186/1475-2859-4-27 PMC1224866 16156893

[B13] Gil-Garcia M. Navarro S. Ventura S. (2020). Coiled-coil Inspired Functional Inclusion Bodies. Microb. Cel Fact. 19, 117. 10.1186/s12934-020-01375-4 PMC7268670 32487230

[B14] Gil-Garcia M. Ventura S. (2021). Coiled-coil Based Inclusion Bodies and Their Potential Applications. Front. Bioeng. Biotechnol. 9, 734068. 10.3389/fbioe.2021.734068 34485264 PMC8415879

[B15] Gorman M. W. Feigl E. O. Buffington C. W. (2007). Human Plasma ATP Concentration. Clin. Chem. 53 (2), 318–325. 10.1373/clinchem.2006.076364 17185366

[B16] Harkness R. A. Coade S. B. Webster A. D. B. (1984). ATP, ADP and AMP in Plasma from Peripheral Venous Blood. Clinica Chim. Acta 143, 91–98. 10.1016/0009-8981(84)90216-X 6096041

[B17] Jäger V. D. Lamm R. Küsters K. Ölçücü G. Oldiges M. Jaeger K.-E. (2020). Catalytically-active Inclusion Bodies for Biotechnology-General Concepts, Optimization, and Application. Appl. Microbiol. Biotechnol. 104, 7313–7329. 10.1007/s00253-020-10760-3 32651598 PMC7413871

[B18] Köszagová R. Nahálka J. (2020). Inclusion Bodies in Biotechnology. Jmbfs 9, 1191–1196. 10.15414/jmbfs.2020.9.6.1191-1196

[B19] Krauss U. Jäger V. D. Diener M. Pohl M. Jaeger K.-E. (2017). Catalytically-active Inclusion Bodies-carrier-free Protein Immobilizates for Application in Biotechnology and Biomedicine. J. Biotechnol. 258, 136–147. 10.1016/j.jbiotec.2017.04.033 28465211

[B20] Kumari S. AmitJamwal R. Jamwal R. Mishra N. Singh D. K. (2020). Recent Developments in Environmental Mercury Bioremediation and its Toxicity: A Review. Environ. Nanotechnology, Monit. Manag. 13, 100283. 10.1016/j.enmm.2020.100283

[B21] Lorenzo-Orts L. Hohmann U. Zhu J. Hothorn M. (2019). Molecular Characterization of chad Domains as Inorganic Polyphosphate-Binding Modules. Life Sci. Alliance 2, e201900385. 10.26508/lsa.201900385 31133615 PMC6537752

[B22] Martinez J. Truffault V. Hothorn M. (2015). Structural Determinants for Substrate Binding and Catalysis in Triphosphate Tunnel Metalloenzymes. J. Biol. Chem. 290, 23348–23360. 10.1074/jbc.M115.674473 26221030 PMC4641920

[B23] Maruthamuthu M. k. Ganesh I. Ravikumar S. Hong S. H. (2015). Evaluation of zraP Gene Expression Characteristics and Construction of a lead (Pb) Sensing and Removal System in a Recombinant escherichia Coli. Biotechnol. Lett. 37, 659–664. 10.1007/s10529-014-1732-x 25433463

[B24] Maruthamuthu M. K. Nadarajan S. P. Ganesh I. Ravikumar S. Yun H. Yoo I.-k. (2015). Construction of a High Efficiency Copper Adsorption Bacterial System via Peptide Display and its Application on Copper Dye Polluted Wastewater. Bioproc. Biosyst. Eng. 38, 2077–2084. 10.1007/s00449-015-1447-y 26219270

[B25] Maruthamuthu M. K. Selvamani V. Nadarajan S. P. Yun H. Oh Y.-K. Eom G. T. (2018). Manganese and Cobalt Recovery by Surface Display of Metal Binding Peptide on Various Loops of OmpC in escherichia Coli. J. Ind. Microbiol. Biotechnol. 45, 31–41. 10.1007/s10295-017-1989-x 29185080

[B26] Mita M. Ito M. Harada K. Sugawara I. Ueda H. Tsuboi T. (2019). Green Fluorescent Protein-Based Glucose Indicators Report Glucose Dynamics in Living Cells. Anal. Chem. 91, 4821–4830. 10.1021/acs.analchem.9b00447 30869867

[B27] Nahalka J. Hrabarova E. (2021). Prebiotic Peptides Based on the Glycocodon Theory Analyzed with FRET. Life 11, 380. 10.3390/life11050380 33922417 PMC8146917

[B28] Nahalka J. Liu Z. Chen X. Wang P. G. (2003). Superbeads: Immobilization in "sweet" Chemistry. Chem. Eur. J. 9, 372–377. 10.1002/chem.200390038 12532285

[B29] Nahálka J. Mislovičová D. Kavcová H. (2009). Targeting Lectin Activity into Inclusion Bodies for the Characterisation of Glycoproteins. Mol. Biosyst. 5, 819–821. 10.1039/b900526a 19603115

[B30] Nahalka J. Nidetzky B. (2007). Fusion to a Pull-Down Domain: a Novel Approach of producingTrigonopsis variabilisD-Amino Acid Oxidase as Insoluble Enzyme Aggregates. Biotechnol. Bioeng. 97, 454–461. 10.1002/bit.21244 17089401

[B31] Nahálka J. Pätoprstý V. (2009). Enzymatic Synthesis of Sialylation Substrates Powered by a Novel Polyphosphate Kinase (PPK3). Org. Biomol. Chem. 7, 1778–1780. 10.1039/b822549b 19590770

[B32] Nahálka J. Vikartovská A. Hrabárová E. (2008). A Crosslinked Inclusion Body Process for Sialic Acid Synthesis. J. Biotechnol. 134, 146–153. 10.1016/j.jbiotec.2008.01.014 18313163

[B33] O'Neill W. C. Sigrist M. K. McIntyre C. W. (2010). Plasma Pyrophosphate and Vascular Calcification in Chronic Kidney Disease. Nephrol. Dial. Transplant. 25, 187–191. 10.1093/ndt/gfp362 19633093 PMC4326300

[B34] Ölçücü G. Klaus O. Jaeger K.-E. Drepper T. Krauss U. (2021). Emerging Solutions for *In Vivo* Biocatalyst Immobilization: Tailor-Made Catalysts for Industrial Biocatalysis. ACS Sust. Chem. Eng. 9, 8919–8945. 10.1021/acssuschemeng.1c02045

[B35] Shi L. Li Y. Liu Z.-P. James T. D. Long Y.-T. (2012). Simultaneous Determination of Hg(II) and Zn(II) Using a GFP Inspired Chromophore. Talanta 100, 401–404. 10.1016/j.talanta.2012.07.097 23141355

[B36] Sorenson A. E. Schaeffer P. M. (2020). A New Bivalent Fluorescent Fusion Protein for Differential Cu(II) and Zn(II) Ion Detection in Aqueous Solution. Analytica Chim. Acta 1101, 120–128. 10.1016/j.aca.2019.12.017 32029102

[B37] Tokunaga M. Arakawa T. Tokunaga Y. Sugimoto Y. Ishibashi M. (2019). Insoluble Expression of Highly Soluble Halophilic Metal Binding Protein for Metal Ion Biosorption: Application of Aggregation-Prone Peptide from Hen Egg white Lysozyme. Protein Expr. Purif. 156, 50–57. 10.1016/j.pep.2019.01.001 30615940

[B38] Villaverde A. Corchero J. L. Seras-Franzoso J. Garcia-Fruitós E. (2015). Functional Protein Aggregates: Just the Tip of the Iceberg. Nanomedicine 10, 2881–2891. 10.2217/nnm.15.125 26370294

[B39] Wang X. Zhou B. Hu W. Zhao Q. Lin Z. (2015). Formation of Active Inclusion Bodies Induced by Hydrophobic Self-Assembling Peptide GFIL8. Microb. Cel Fact 14, 88. 10.1186/s12934-015-0270-0 PMC4467046 26077447

[B40] Wu W. Xing L. Zhou B. Lin Z. (2011). Active Protein Aggregates Induced by Terminally Attached Self-Assembling Peptide ELK16 in escherichia Coli. Microb. Cel Fact 10, 9. 10.1186/1475-2859-10-9 PMC3045283 21320350

[B41] Zhang X. Hu Y. Yang X. Tang Y. Han S. Kang A. (2019). FÖrster Resonance Energy Transfer (FRET)-based Biosensors for Biological Applications. Biosens. Bioelectron. 138, 111314. 10.1016/j.bios.2019.05.019 31096114

[B42] Zhou B. Xing L. Wu W. Zhang X.-E. Lin Z. (2012). Small Surfactant-like Peptides Can Drive Soluble Proteins into Active Aggregates. Microb. Cel Fact. 11, 10. 10.1186/1475-2859-11-10 PMC3398302 22251949

