# Supplementary material for: Pull-Down Into Active Inclusion Bodies and Their Application in the Detection of (Poly)-Phosphates and Metal-Ions
Source: Front Bioeng Biotechnol. 2022 Mar 1;10:833192. doi: 10.3389/fbioe.2022.833192 (PMC8921494; doi:10.3389/fbioe.2022.833192)
Supplement: Supplementary file 1 [file DataSheet1.PDF]

frontiers

Supplementary information

Pull-down into active inclusion bodies and their application in the detection of (poly)-phosphates and metal-ions

Eva Hrabarova[1,2], Martina Belkova[1,2], Romana Koszagova[1,2] and Jozef Nahalka[1,2,*]

[1] Institute of Chemistry, Centre for Glycomics, Slovak Academy of Sciences, Dubravska cesta 9, SK-84538 Bratislava, Slovak Republic

[2] Institute of Chemistry, Centre of excellence for white-green biotechnology, Slovak Academy of Sciences, Trieda Andreja Hlinku 2, SK-94976 Nitra, Slovak Republic.

*** Correspondence:**

Jozef Nahalka

nahalka@savba.sk

1 Supplementary figure

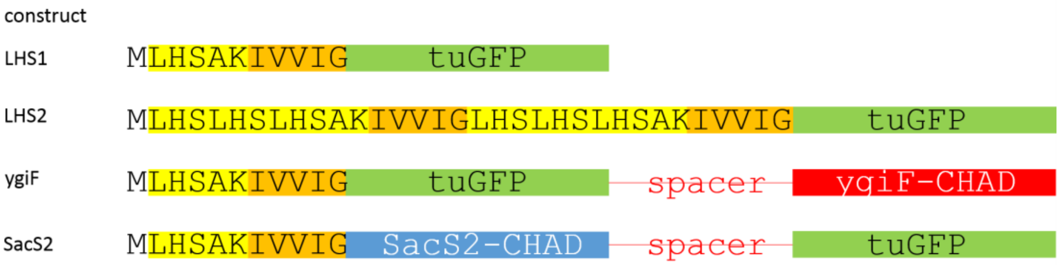

Figure 1

The graphical illustrations of the constructs.

2 Supplementary sequence information

```
>sp|P30871|3PASE_ECOLI Inorganic triphosphatase OS=Escherichia coli (strain K12)
OX=83333 GN=ygiF PE=1 SV=1
```

<u>CHAD domain of ygiF$^{221-422}$</u>

```
MAQEIELKFIVNHSAVEALRDHLNTLGGEHHDPVQLLNIYYETPDNWLRGHDMGLRIRGENGRYEMTMKVAGRVTGGLHQR
PEYNVALSEPTLDLAQLPTEVWPNGELPADLASRVQPLFSTDFYREKWLVAVDGSQIEIALDQGEVKAGEFAEPICELELE
LLSGDTRAVLKLANQLVSQTGLRQGSLSKAARGYHLAQGNPAREIKPTTILHVAAKADVEQGLEAALELALAQWQYHEELW
VRGNDAAKEQVLAAISLVRHTLMLFGGIVPRKASTHLRDLLTQCEATIASAVSAVTAVYSTETAMAKLALTEWLVSKAWQP
```

FLDAKAQGKISDSFKRFADIHLSRHAAELKSVFCQPLGDRYRDQLPRLTRDIDSILLLAGYYDPVVAQAWLENWQGLHHAI
ATGQRIEIEHFRNEANNQEPFWLHSGKR

>tr|Q97YW1|Q97YW1_SACS2 Uncharacterized protein OS=Saccharolobus solfataricus (strain ATCC 35092 / DSM 1617 / JCM 11322 / P2) Sulfolobus solfataricusOX=273057 GN=SSO1190 PE=4 SV=1 UniProtKB - Q97YW1 (Q97YW1_SACS2)

MTISQVKDYLNLQLKKAIQINGIGVEEIHDMRVAVRKYFDVLYAIHPVYENVECLFLAKEAIKRLGKVRDMDICEIANGER
TKLAIRALKDVRELQVCFVNDKIYGVRLTIYNRILSSLHQIQDITDFHELRKNIRVTRNLVEALGYDNTEIKALAKKMGDI
RDEILKMRCRGLTPPDINIIQYKEEAKRVILKIIASQEEFHHFKIEDRY

>LHS1 construct

MLHSAKIVVIGLPAMEIECRITGTLNGVEFELVGGGEGTPEQGRMTNKMKSTKGALTFSPYLLSHVMGYGFYHFGTYPSGY
ENPFLHAINNGGYTNTRIEKYEDGGVLHVSFSYRYEAGRVIGDFKVMGTGFPEDSVIFTDKIIRSNATVEHLHPMGDNDLD
GSFTRTFSLRDGGYYSSVVDSHMHFKSAIHPSILQNGGPMFAFRRVEEDHSNTELGIVEYQHAFKTPD*

>LHS2 construct

MLHSLHSLHSAKIVVIGLHSLHSLHSAKIVVIGLPAMEIECRITGTLNGVEFELVGGGEGTPEQGRMTNKMKSTKGALTFS
PYLLSHVMGYGFYHFGTYPSGYENPFLHAINNGGYTNTRIEKYEDGGVLHVSFSYRYEAGRVIGDFKVMGTGFPEDSVIFT
DKIIRSNATVEHLHPMGDNDLDGSFTRTFSLRDGGYYSSVVDSHMHFKSAIHPSILQNGGPMFAFRRVEEDHSNTELGIVE
YQHAFKTPD*

>LHS1-GFP-ygiF construct

MLHSAKIVVIGLPAMEIECRITGTLNGVEFELVGGGEGTPEQGRMTNKMKSTKGALTFSPYLLSHVMGYGFYHFGTYPSGY
ENPFLHAINNGGYTNTRIEKYEDGGVLHVSFSYRYEAGRVIGDFKVMGTGFPEDSVIFTDKIIRSNATVEHLHPMGDNDLD
GSFTRTFSLRDGGYYSSVVDSHMHFKSAIHPSILQNGGPMFAFRRVEEDHSNTELGIVEYQHAFKTPDIKPTTILHVAAKA
DVEQGLEAALELALAQWQYHEELWVRGNDAAKEQVLAAISLVRHTLMLFGGIVPRKASTHLRDLLTQCEATIASAVSAVTA
VYSTETAMAKLALTEWLVSKAWQPFLDAKAQGKISDSFKRFADIHLSRHAAELKSVFCQPLGDRYRDQLPRLTRDIDSILL
LAGYYDPVVAQAWLENWQGLHHAIATGQRIEIEHFRNEANNQEPFWLHSGKR*

>LHS1-SACS2-GFP construct
MLHSAKIVVIGISQVKDYLNLQLKKAIQINGIGVEEIHDMRVAVRKYFDVLYAIHPVYENVECLFLAKEAIKRLGKVRDMD
ICEIANGERTKLAIRALKDVRELQVCFVNDKIYGVRLTIYNRILSSLHQIQDITDFHELRKNIRVTRNLVEALGYDNTEIK
ALAKKMGDIRDEILKMRCRGLTPPDINIIQYKEEAKRVILKIIASQEEFHHFKIEDRYIKPTTILHVAAKADLPAMEIECR
ITGTLNGVEFELVGGGEGTPEQGRMTNKMKSTKGALTFSPYLLSHVMGYGFYHFGTYPSGYENPFLHAINNGGYTNTRIEK
YEDGGVLHVSFSYRYEAGRVIGDFKVMGTGFPEDSVIFTDKIIRSNATVEHLHPMGDNDLDGSFTRTFSLRDGGYYSSVVD
SHMHFKSAIHPSILQNGGPMFAFRRVEEDHSNTELGIVEYQHAFKTPD*
